# Ethical Foundations of the Accompanying Patient’s Role for an Enhanced Patient Experience: A Scoping Review

**DOI:** 10.3390/jpm13010077

**Published:** 2022-12-29

**Authors:** Mylène Shankland, Amaryllis Ferrand, Isabelle Ganache, Marie-Andrée Côté, Marie-Pascale Pomey

**Affiliations:** 1 Bioethics Program, Department of Social and Preventive Medicine, School of Public Health, Université de Montréal, Montréal, QC H3N 1X9, Canada; 2 Pragmatic Health Ethics Research Unit, Montreal Clinical Research Institute (IRCM), Université de Montréal, Montréal, QC H2W 1R7, Canada; 3 Independent Researcher, Montréal, QC H2X 0A9, Canada; 4 Department of Health Management, Evaluation and Policy, School of Public Health, Université de Montréal, Montréal, QC H3N 1X9, Canada; 5 CHUM Research Centre, Health Innovation and Evaluation Hub, Montréal, QC H2X 0A9, Canada

**Keywords:** ethics, ethical foundations, patient advisor, care partnership, accompanying patient, peer support, patient partner

## Abstract

In recent years, recognizing patients’ experiential knowledge to improve the quality of care has resulted in the participation of patient advisors at various levels of healthcare systems. Some who are working at the clinical level are called accompanying patients (AP). A PRISMA-ScR exploratory scoping review of the literature was conducted on articles published from 2005 to 2021. Articles not in English or French and grey literature were excluded. The databases searched included Medline, PubMed, Scopus, and Google Scholar. The data were organized according to the similarities in the ethical foundations of the included papers. Out of 2095 identified papers, 8 met inclusion criteria. Terms used to describe APs included peer support, resource parent, and peer health mediator. The clinical settings included psychiatry/mental health and neonatology. APs, patients, healthcare professionals, managers and policy makers were included in the studies. Three personal ethical foundations describing the foundations of the AP role were found: resilience, listening skills and altruism. The ethical foundations of this role also addressed interpersonal and interprofessional relationships with other actors in the healthcare system. The literature on the ethical foundations of APs is sparse, with heterogeneous methodologies. Further studies mobilizing well-defined methodologies would further validate the current results and deepen our understanding of the ethical foundations of the AP role.

## 1. Introduction

In recent years, recognizing patients’ experiential knowledge to enhance quality of care has resulted in the creation of the role of patient advisors (PAs). PAs are patients, currently or previously receiving care, participating in various levels of the healthcare system. They are involved in clinical care, research, policy making, and the training of health professionals [1]. At the clinical level, PAs are known as accompanying patient (AP) and they develop experiential knowledge through living with a health condition and receiving healthcare services. They complement the scientific and professional knowledge of caregivers, researchers, and trainers. Inscribed in the Montreal model [2], the care partnerships between an AP, a healthcare team and patients allow patients to invest in their care, make informed decisions based on their life plans and become their own caregivers. At the clinical level, the AP role is rooted in a desire to mobilize APs’ expertise as patients beyond their own care, to provide emotional, informational, and educational support to other patients who are experiencing situations that they may have experienced themselves. APs complement the services offered by healthcare professionals by providing additional and unique expertise stemming from their personal experiences in the healthcare system. The particularity of the AP role is that an AP becomes a full member of the team, developing interpersonal and interprofessional relationships with all parties in the healthcare network. However, the arrival of this new actor in clinical teams raises certain ethical questions, particularly regarding integration, contribution and recognition, in addition to issues of confidentiality and the loyalty conflicts that arise when exercising this role [3,4]. Researchers have addressed some of the ethical issues underlying the introduction of APs at different levels of the healthcare system, including highlighting emerging ethical issues [1,4,5,6] such as remuneration, tokenism, and the professionalization of this role [4]. These issues call for work to clarify and define the ethical foundations of the emerging AP role and of its position within the clinical team. In order to bring forth the ethical foundations of the role of AP, we intend to describe the principles, values, and motivations that guided APs toward the healthcare system and encouraged them to become involved and help other patients living with a similar health condition [7]. This is a skill set of intrinsic know-how competencies based on who APs are and what they needed to mobilize to be part of a clinical team, including the organizational imperative of healthcare systems. APs’ ethical foundation may be compared to the professional conduct expected by employees/staff members in healthcare systems.

We conducted an exploratory scoping review of the ethical foundations underlying the AP role. This review is part of an overarching project, PAROLE-Onco (Le Patient Accompagnateur, une Ressource Organisationnelle comme Levier pour une Expérience patient améliorée en oncologie/[The Accompanying Patient, an Organizational Resource as a Lever for an Enhanced Oncology Patient Experience]), which seeks to better understand the concept of patient advocates as integral members of clinical teams and their impact on the quality and safety of care. These caregivers are patients who have experienced a health condition and are sharing their experience and experiential knowledge to the benefit of other patients with the same health problem.

## 2. Materials and Methods

The Preferred Reporting Items for Systematic Reviews and Meta-Analyses extension for scoping review (PRISMA-ScR) checklist was used as a guideline to ensure the methodological transparency of this review [8]. This scoping review was conducted according to the Arksey and O’Malley framework [9]. The framework consists of the following six stages: (1) identifying the research questions, (2) identifying relevant studies, (3) selecting the studies, (4) charting the data, (5) collating and summarizing, and (6) reporting the results.

### 2.1. Research Questions

This scoping review was based on the following research questions:

- What are the motivations and the reasons for which accompanying patients become involved with other patients with a similar health problem?
- What is the nature of the relationship between accompanying patients and various other actors in the healthcare system (i.e., the patients advised, the health professionals and the health organizations)?

### 2.2. Identifying Relevant Studies

The following eligibility criteria were established to guide the literature review. We included articles published from 2005 to 2021 to reflect the recent nature of patient partnership. This time period was selected because partnership models with patient-centered approaches to care emerged in the early 2010s [2].

We included publications in English and French whose designs produced primary empirical and theoretical studies (e.g., quantitative, qualitative, or mixed methods) that were published in the peer-reviewed literature. Publications were excluded if they were considered grey literature (e.g., reports, theses, newsletters). The target population was accompanying patients. 

The studies eligible for inclusion were those in which the AP was involved with both patients and healthcare personnel at the same time and for the same purpose. Articles on patient engagement in their own care, decision-making, research, and training were excluded. 

Medline, PubMed, Scopus and Google Scholar databases were used to capture the literature published in the medical and social science domains. These databases were selected to effectively capture an extended range of literature and to avoid literature in disciplines irrelevant to the topic. The search strategy was developed in Ovid Medline (Box 1). It consisted of keywords and subject headings. It was then adapted for use with other databases. The electronic databases were searched in April 2021.

Box 1 **Search Strategy in Ovid Medline**

Ethics, Medical/ OR Ethics, Clinical/ OR Ethics/ OR Ethics, Nursing/ OR Ethics, Pro-fessional/ OR Ethics, Institutional/ OR “Attitude of Health Personnel”/

 

  AND

 

Patient participation/ OR patient engagement OR patient partner OR patient re-source OR patient expert OR patient advisor

### 2.3. Study Selection

**Figure 1:**
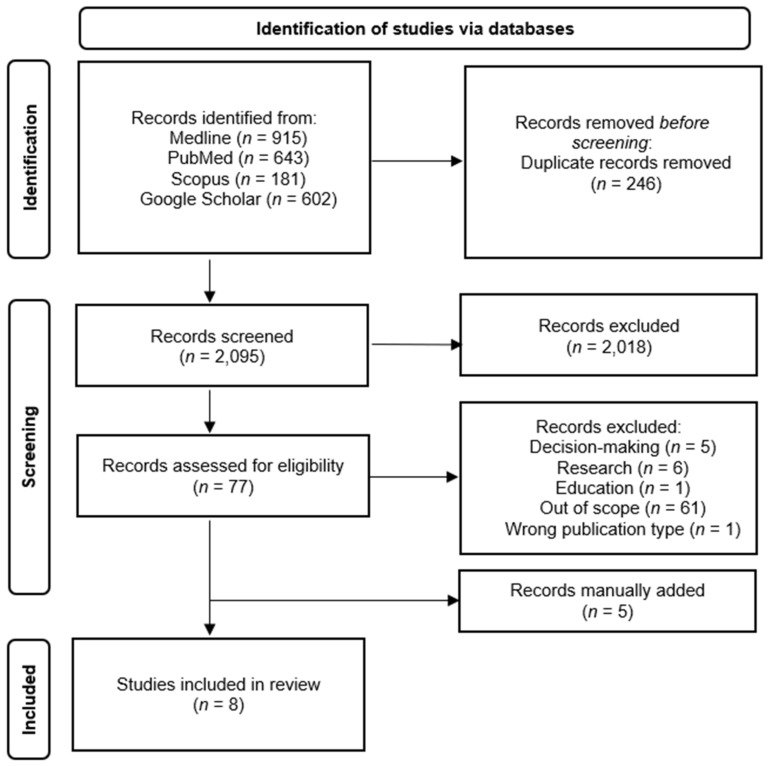
PRISMA Diagram. From: Page MJ, McKenzie JE, Bossuyt PM, Boutron I, Hoffmann TC, Mulrow CD et al. The PRISMA 2020 statement: an updated guideline for reporting systematic reviews. BMJ 2021;372:n71. doi:10.1136/bmj.n [10].

After duplicates were removed, a total of 2095 articles titles and abstracts were assessed for eligibility by the first author (MS) and 20% of them were reviewed by the second author (AF). The screening process is detailed in the PRISMA flow diagram shown in Figure 1.

### 2.4. Charting the Data

An inductive data extraction chart was developed based on an initial analysis of 25% of the data. The chart was further refined and validated with a multidisciplinary team of researchers in public health, medicine, ethics, and sociology. One reviewer collected the data (MS) and another reviewer (AF) double-checked 20% of the data collected. A third senior reviewer (IG) was consulted as needed to confirm the charting process. The following data were extracted from the selected articles:

- Descriptive characteristics (e.g., author, year, country, publication date, setting, study aim, study design, participants’ characteristics, and the term used to describe accompanying patients) were collected;
- Relevant results were organized on AP involvement at various levels (e.g., the personal, clinical and systemic levels);
- The data were organized according to similarities in the ethical foundations discussed in the articles.

The methodological quality of the studies was assessed using the 2018 version of the Mixed Method Appraisal Tool (MMAT) [11].

### 2.5. Collating, Summarizing, and Reporting the Results

This scoping review mobilized a narrative sociological approach that provides a comprehensive view of the value added by accompanying patients in the ecosystem of a healthcare system. This approach is supported by the link between patients and APs, based on connections with respect to the series of events that constitute their lived experience in various structures [12]. Consequently, we focus our reading on the narrative role of APs in the relational and social domains, taking into consideration the dynamic between professional and personal perspectives [12]. We chose this approach because the role of AP is halfway between a helping and a caring relationship [13]. Acting on several levels within institutions [12], we noted, based on the literature, the AP’s interventions in the relational chain of the hospital environment [14]. The nature of the links with healthcare personnel and the environment is a determining factor in the perceived quality of the services obtained [14,15]. We therefore explore APs’ various levels of influence to identify the ethical foundations of their involvement and to demonstrate the relevance of integrating APs into clinical teams and the healthcare system. 

## 3. Results

Our results include the characteristics of this study and findings related to our research questions. 

### 3.1. Article Methodologies and Population (Table 1)

The articles were published in the period 2015 to 2019 and represented a total of 7 studies, with 1 study being published in 2 articles [16,17]. Various methodologies were used in 5 empirical qualitative studies [13,16,17,18,19,20], including a study published in 2 articles, 1 empirical mixed methodology [21] and 1 review, which based their arguments on literature and personal experiences as healthcare professionals practicing with APs [22]. These studies were conducted in four countries: 4 in Canada [13,19,21,22], 2 in France [16,17], 1 in Norway and Canada [18] and 1 in the United Kingdom [20]. As for the language of publication, 4 studies were in English (4 studies) [18,20,21,22] and 3 were in French (including the study published in 2 articles) [13,16,17,19].

Four terms were used to describe what we called the accompanying patients. The most-used term was a variation of peer support with “pair aidant [peer supporter]” (*n* = 2) [13,19], “peer support workers” (*n* = 1) [20] and “peer support provider” (*n* = 1) [18] followed by “resource parent” because of the specific medical area of those studies (*n* = 2) [21,22] and “médiateur santé pair [peer health mediator]” (*n* = 1) [16,17].

The practice settings where the studies were conducted were psychiatry/mental health (*n* = 5) [13,16,17,18,19,20] and neonatology (*n* = 2) [21,22].

This review included the perspectives of patients [16,17,18,19,21], APs [13,16,17,18,19,21], healthcare professionals [18,19,20,21,22], healthcare managers [18,19] and policy makers [18]. 

### 3.2. Ethical Foundations Associated with the Personal Characteristics of Accompanying Patients

Three ethical foundations were identified in 6/8 papers to highlight the key personal ethical foundations associated with APs: resilience [13,19,21,22], listening skills [16], and altruism [18,21] (Table 2).

### 3.3. Foundations of the Relationship (Table 3)

#### 3.3.1. With a Patient

We found 5 ethical foundations in 7/8 papers concerning the relationship with the patient, including hope [13,16,17,18,19,20,22], a reciprocal and egalitarian relationship with the advised patient [13,16,18,20], the autonomy of the patient advised [18], support [16,17,20], and real empathy [13,18,19,20]. 

#### 3.3.2. With the Clinical Team

We found 6 ethical foundations in 6/8 papers concerning the relationship with the clinical team, including complementarity [18,20,21,22], collaboration [16,18,19,22], assertiveness [19,20], openness [19], respect [19], and respect of privacy [16]. 

#### 3.3.3. With the Healthcare Setting

We found 6 ethical foundations in 6/8 papers used to describe the AP’s relationship with the healthcare setting: commitment [13,18,19,20,21,22], responsibility [13,18,19,20,22], versatility [13,19,20,22], recognition [13,22], health democracy [19], and transparency [13].

#### 3.3.4. With the Patient and the Clinical Team

We found 2 ethical foundations in 3/8 papers concerning the relationship with the patient and the clinical team: confidentiality [13,18], and professionalism [16].

#### 3.3.5. With the Clinical Team and the Healthcare Setting

We found 1 ethical foundation in 1/8 paper concerning the relationship with both the clinical team and the healthcare setting: collaboration [17].

#### 3.3.6. With the Healthcare Setting and Society 

We found 1 ethical foundation in 2/8 papers concerning the relationship with both the healthcare setting and society: collaboration [13,22].

## 4. Discussion

Over the last few decades, new roles and the involvement of patients in healthcare systems have led to the development of the AP role. While models such as the Montreal model have led the way to integrating these helping patients into clinical teams and healthcare settings [2], there are still unresolved ethical questions regarding this role. To address these challenges, there is a need to understand the ethical foundations of the AP role.

To our knowledge, this scoping review is the first to explore the ethical foundations of APs. It has revealed the underlying ethical foundations and there is a paucity of literature specifically addressing this emerging role. The studies were mostly conducted in Canada [13,18,19,21,22] and France [16,17], with only two conducted in Norway [18] and the United Kingdom [20]. 

The variety of vocabulary used demonstrates the lack of consensus around a term to describe the AP role and the influence of practice settings. In neonatology, the term *resource parent* is favoured [21,22]. This refers specifically to the parent role in their involvement with other health service users. Additionally, the variety of terms is symptomatic of the level of institutionalization of the AP role. The term “peer health mediator” is used specifically in France [16,17] and is directly linked to an institutionalized program of study [23]. Additionally, the Canadian mental health field favours the term “peer support” [13,18,19,20], which is a practice that has historically taken place outside of care settings.

Some authors have identified peer support values as “hope and recovery, self-determination, empathic and equal relationships, dignity, respect and social inclusion, integrity, authenticity and trust, health and wellness, lifelong learning and personal growth” [18] (p.68). This corroborates the proximity between peer support and the AP role and supports the results of this review. 

### 4.1. Who Are the APs?

The foundations intrinsic to APs suggest the personal characteristics embedded in peer support practices. This role is merged with the common partnership model, known as the implication of patients in care, policies, research, and training, to motivate others to self-determination [18,23]. APs activate the key principles of partnership in other patients by using their experiential knowledge to improve well-being and motivate others to be part of their own care. 

The involvement of APs enters into a logic of social exchange associated with selflessness [24], real empathy [13,18], destigmatizing illness [19,20] and encouraging patients to hope for recovery [13,16,17,18,19,20,22]. Embodying the hope of recovery, the AP is the materialization of the benefits and outcomes of the patient’s treatments or interventions. Indeed, the uncertainty of care processes and success is expected, but the presence of the AP serves to demonstrate that the care provided can work. This reinforces the patient’s confidence in the clinical team, the healthcare system and the care promulgated [25]. In this way, APs improve the relational chain of care within the healthcare system [14], thereby increasing the perceived quality of the care received [25]. In this sense, we support the idea that the form of exchange matters as much as its content.

### 4.2. What Is the Added Value of AP?

Mobilizing a personal experience of their life with illness, APs transform their knowledge into practical and technical tools to support patients. Listening skills and authenticity make APs more approachable and give patients the opportunity to share personal thoughts outside of their health concerns [17,20]. 

The complementarity and collaboration with the clinical team improves services [18,20,21]. As some professionals could perceive the addition of this new member as a failure to develop a professional relationship with patients and an overlap with their responsibilities [18,21], the AP role must be well defined by clearly differentiating the tasks and expectations for all concerned [13,18,19,20,21,22]. In this regard, healthcare settings have a responsibility to properly integrate APs, thereby ensuring their well-being, just as they do for staff members [13,20,22]. 

The unique perspective of APs enables health democratization [23] and full citizenship [19], since they symbolize the accessibility of health to everyone outside the healthcare system [23], notably because their knowledge differs from the theoretical outcomes [16].

### 4.3. What Are the Challenges for Institutionalization?

From a management perspective, healthcare systems play a leading role in the deployment and ethical support of APs. The literature presents a variety of models, including some in which APs take specific training within the education system and receive a salary [16,17,20,23], while in others, they suggest training from the healthcare institution and compensation can still be a thorny issue [13,18,19,21,22]. Some authors suggest that remuneration denatures the foundational AP role [13,22] and removes their independent voice [21]. However, our colleagues have analyzed the legal considerations around AP integration in the Québec, Canada healthcare system and concluded that, with or without remuneration, APs still have allegiance to the system [26]. Therefore, without any compensation—beyond being reimbursed for costs related to parking or public transportation, lunch, etc.—the lack of recognition is noticeable and does not capture a full picture of the allegiance dilemma.

Otherwise, APs in the PAROLE-Onco project have raised another ethical dilemma regarding allegiance. Regardless of their allegiance to the system, they may sometimes be in situations that make them feel that they must take the side of either the patient or the clinical team. At this time, this question has not been resolved, but it gives a sense of how allegiance can pose challenges and suggests that we need to take this ethical concern seriously.

This review has some limitations. Few studies were found on this subject. The lack of results demonstrates a gap in the literature that needs to be filled. Restricting the searches to English and French may have excluded relevant research published in other languages. Considering the variety of terms used to describe APs, it is possible that other terms, unknown to our research team, are also used for this role.

## 5. Conclusions

Even though we found few studies and little diversity in the methodologies used in the field, some more foundations of the AP role were identified, including resilience, listening skills and altruism. Future studies mobilizing stronger methodologies would help us understand how these foundations are applied in practice and how the results obtained in this review reflect APs’ experiences in healthcare systems.

We found that psychiatry/mental health and neonatology are two medical domains that directly address the ethical foundations of the AP role. Both fields engage in peer support and have demonstrated the applicability and added value of integrating APs into clinical teams and healthcare systems. The extant literature on ethics and patient partnership is more focused on research and training issues. However, the values and dilemmas seem to be quite similar among the various partnership models. In this regard, the next literature review should focus on the overall partnership instead of its separate areas.

More empirical studies are needed to further describe the values of the emerging AP role in order to better circumscribe the ethical foundations of this role.

## Figures and Tables

**Table 1:** Characteristics of the studies on the ethical foundation of the AP role.

Authors and Year of Publication	Country(ies)	Aims of the Study	Methodologies Used	Populations and Sizes Studied	Terms Used
Lierville, A.-L. et al. (2015) [13]	Canada	“To question both the issues related to the practice of peer support workers and patient partners and those related to the support mechanisms put in place by the organization to support these new workers and the environments in which they operate” [13] (p.119)	Qualitative Interviews	15 peer supporters	Pair aidant [peer supporter]
Demaily, L. and Garnoussi, N. (2015a) [16] and (2015b) [17]	France	To address the encounters between psychiatric users and new professionals, peer health mediators, trained in a French experimental program, with a sociological lens [16,17]	Qualitative Ethnography Interviews	74 patients Unknow numbers of peer health mediators	Médiateur santé pair [peer health mediator]
Mulvale, G. et al. (2019) [18]	Canada, Norway	To “focus on the perceived value of peer support and the strategies used to support [peer support provider] integration in clinical settings based on a case study of mental health peer support programs in Ontario and Norway” [18] (p.69)	Qualitative Case study Focus group (peer support providers) Interviews (other participants)	9 peers 33 peer support providers 20 managers/health team members 6 policy/decision makers	Peer support provider
Bourque, C.J. et al. (2018) [22]	Canada	To “describe and [to] examine recent experiences regarding the involvement of resource parents in neonatology, based on their experience in CHU Sainte-Justine (Montréal) and in Sunnybrook Hospital (Toronto) and on the current literature.” [22] (p.45) “To define what a resource parent is, what activities they currently do in neonatology and how these activities can be classified.” [22] (p.45)	Lived experience of the authors Literature review	Review	Resource parent
Goulet, M.-H. et al. (2018) [19]	Canada	“To explore the feasibility, acceptability and the impact of post-isolation return on mental health with the support of a peer support worker” [19] (p.42)	Qualitative Interviews Focus group Self-administered Survey	6 members of the different management 9 clinical members 5 patients 1 peer support	Pair aidant [peer supporter]
Collins et al. (2016) [20]	United Kingdom	“To gain insight into the views and attitudes psychiatrists have about [peer Support workers].” [20] (p.279)	Qualitative Interviews	11 psychiatrists	Peer support workers
Dahan, S. et al. (2018) [21]	Canada	“To analyze activities involving veteran resource parents and patients in a family partnership program; their perspectives were also explored” [21] (p.123) “To examine the multiple roles assumed by these family stakeholders and describe the impact of their integration in different initiatives” [21] (p.123)	Mixed methodology Quality control Questionnaire with open-ended questions	28 resource parents 2 patients 27 providers	Veteran resource parent

**Table 2:** Personal ethical foundations of accompanying patients.

Foundation	Description	Example
Resilience	Fragility related to reliving a traumatic experience and working with it	“Three parents reported more significant impacts: reliving a traumatic experience during a simulation scenario” [21] (p. 126)
Listening skills	Ability and time to discuss personal life issues, outside health purposes, that affect the patient’s well-being	“One of the differential qualities of the mediator lies in his availability and more generally in his accessibility: the quantitative dimension of the time he can devote to individuals is generally associated with the ease of entering into a relationship with him, or with the ease with which he allows communication.” [16] (p. 177)
Altruism	To contribute to the health care system after receiving so much from it	“At the present time in neonatology, when parents become involved as resource parents, they are often either integrated in a project or a committee in a non-official way. Some parents, such as Robin, directly seek a way to help or ‘give back,’ either through parental associations or by communicating their desire to providers they are still in contact with.” [22] (p. 45)

**Table 3:** Ethical foundations of the role of accompanying patients based on relationships.

Foundation	Description	Example
Relationship with the patient		
Hope	Be seen as a model of recovery	“The only consistent thought between all the interviewees was that [peer support workers] would use their lived experience of mental illness to support service users, some suggesting this would inspire hope” [20] (p. 281)
Reciprocity	Have an equal relationship with the patient	“Many referred to [peer support workers] as having a kind of authenticity; as being more approachable, and therefore able to offer something different from other staff” [20] (p. 280)
Autonomy	Activate the autonomy of others by encouraging the patient to be a partner in their own treatment	“Peer support workers work to foster peer independence in decision-making” [18] (p. 70)
Support	Accompany the patient in their life after their health issues	“The second process is meeting a non-shaming person and a hopeful role model: the peer health mediator functions as a positive support to identify with. This is someone who has come out of it, who has found a job, for example.” [17] (p. 191)
Real empathy	Sincerely know what it is to live with a health issue, based on their lived experience	“We also note that, according to the majority of the people we met, only or almost only peer support workers seem to be able to have a real empathy” [13] (p. 131)
Relationship with the clinical team		
Complementarity	Add value to the clinical team	“The majority (90%) described advantages related to their collaborations with resource parents and patients, invoking [notably]: (1) the resource parent’s and patient’s complementary roles […]; (2) the resource parent’s stories as essential to improve caring and communication […]” [21] (p. 126)
Collaboration	Understand the roles of each contributor	“[the peer supporter] therefore made sure that his feedback with the clinical team was as synthetic as possible, always with a view to helping them better understand the patient’s experience” [19] (p. 46)
Assertiveness	Be able to assert the relevance of the AP role while respecting the skills of the clinical team	“For the peer support worker, concerns are raised about the difficulties inherent in the intensive care setting (people at risk of aggression) and the feeling of having to defend a new professional role.” [19] (p. 45)
Openness	Be responsive to the addition of a new member	“On the stakeholders’ side, there is an openness to new ways of improving” [19] (p. 44)
Respect	Be able to separate the opinions of the patients from the real qualities of the clinical team	“The clinical team stressed the importance of creating an atmosphere where the provider does not feel judged. Some providers said they felt uncomfortable not being able to contextualize what the patient was saying as reported by the AP.” [19] (p. 46)
Respect of privacy	Separate the clinical team’s private information from information relevant to the patient’s care	“The de-cleavage goes so far as to play out also in relation to the “private” life of a caregiver that the peer health mediator “naturally” discussed with a user (which was a source of tension and debate in the team). This relative de-cleavage, which is a technique of relational work, leads reciprocally to more confidence on the part of the user, but confidentiality confidence is not a general and discriminating characteristic of the peer health mediator/patient encounter.” [16] (p. 180)
Relationship with the health organization		
Commitment	Commit to the AP to ensure integration	The “demands need to be clear: what is the project, how long would it last, where would it take place, is there a remuneration or compensation, and what are the potential risks associated with the project?” [22] (p. 49)
Responsibility	Assume responsibility for a new contributor to the organization	“There are higher risk and responsibility stakes for support roles where resource parents meet new parents in vulnerable situations. In these cases, careful guidelines will need to establish boundaries between the responsibility of support parents and those of other providers” [22] (p. 49)
Versatility	Have the skills needed to perform multiple roles and meet a variety of expectations	“[The peer supporters ] find themselves playing a dual role. They are called upon to play the role of a patient in recovery while also being an advocate for other patients receiving services” [19] (p. 49)
Recognition	Recognize the added value of APs to health organizations	“Resource parents are also remunerated in some institutions. The question of whether parents should be remunerated—in addition to transportation and meals—is debated. Some parents and investigators consider that this expression of altruism and “giving back” loses its value when it is for sale. On the other hand, for time consuming tasks, not being able to compensate parents for their time may translate to a lack of resource parent participation and limit the diversity of parental voices. It is unclear whether financial compensation leads to negative impacts on parental participation, such as undue coercion of parents” [22] (p. 49)
Health democracy	Symbolize the accessibility of health	“The involvement of a peer supporter in [post-isolation return] was seen as operationalizing the concepts of recovery and full citizenship within the philosophy of reducing [seclusion with restraint].” [19] (p. 44)
Transparency	Be honest and clear about the tasks and expectations of the AP role	“One peer supporter regretted not being able to rely on a clearer job description, function and status.” [13] (p. 128)
Relationship with both the patient and the clinical team		
Confidentiality	Disclose only relevant information to improve patients’ health and well-being	“There are questions, for example, about who does what on clinical teams or committees, whether peers or patient partners are third parties or team members. The resulting technical issues are a problem for many managers we met. They are related to the sharing of confidential information in connection with access to medical records or team meetings.” [13] (p. 129)
Professionalism	Ensure the integrity of the professions of health organization contributors	“As a result of their encounters with [peer health mediators], users feel authorized to make certain criticisms of other professionals or of the health care system. However, [peer health mediators] realize that criticism is not always tenable and they may also be able to explain the institutional constraints” [16] (p. 185)
Relationship with both the clinical team and the healthcare setting		
Collaboration	Understanding the role of each contributor	“The institutionalization of peer health mediators is likely to make them auxiliaries of the professionals. This risk is already suggested by the recurrence of the term ‘professional like any other’, signifying for the peer health mediators or their colleagues a successful integration.” [17] (p. 198)
Relationship with both the healthcare setting and society		
Collaboration	Understanding the roles of each contributor	“Resource parents share a desire to contribute, but their recruitment has not been rigorously investigated and no procedure currently exists to describe how to recruit a veteran parent for a given role. Many foundations and/or parent associations, such as the March of Dimes, Canadian Premature Babies Foundation, Miracle Baby Foundation, or Préma-Québec are contacted by parents who wish they could do more for the cause and for families. Similarly, hospital foundations are also contacted by veteran parents who wish to give back.” [22] (p. 48)

## Data Availability

The dataset supporting the conclusions of this article is included in the article.
